# Non-conventional Genetic Basis of Congenital Adrenal Hypoplasia in South Asia

**DOI:** 10.7759/cureus.23527

**Published:** 2022-03-26

**Authors:** Sudip Chatterjee, Anirban Majumder

**Affiliations:** 1 Department of Endocrinology, Vivekananda Institute of Medical Sciences, Kolkata, IND; 2 Department of Endocrinology, Kali Prasad Chowdhury Medical College and Hospital, Kolkata, IND

**Keywords:** south asia, aaas gene, clinical genomics, genetic basis, congenital adrenal hypoplasia

## Abstract

Congenital adrenal hypoplasia or adrenal hypoplasia congenita (AHC) is a rare disorder ascribed to mutations in three genes, namely, the dosage-sensitive sex reversal-adrenal hypoplasia congenita critical region on the X chromosome, gene 1 (DAX-1/NROB1 gene), steroidogenic factor-1 gene (SF-1/NR5A1 gene), and Achalasia-Addisonianism-Alacrima syndrome gene (AAAS gene). Five cases of AHC of local South Asian origin are described here. Golden Helix VarSeq 2.2.0 (Golden Helix Inc., Bozeman, MT, United States), the clinical genomics interpretation and reporting platform, was used for genetic study. No subject had congenital adrenal hyperplasia (CAH). Four male neonates presented with hypoglycemia, and one older female child presented with hyperpigmentation. This girl had a recognized mutation in the AAAS gene, while none of the four male neonates had any of the recognized mutations associated with AHC. Further, none were salt-losing, which is the conventional Western phenotype. Thus, additional, yet unknown, gene(s) must be operative in AHC among South Asian subsets.

## Introduction

Adrenal insufficiency (AI) is due to either dysfunction of the adrenal gland (primary AI), deficient pituitary adrenocorticotrophic hormone (ACTH) secretion (secondary AI), or deficient hypothalamic corticotropin-releasing hormone (CRH) secretion (tertiary AI). Congenital adrenal hyperplasia (CAH) accounts for 70% of pediatric patients with primary AI [1]. Adrenal hypoplasia congenita (AHC) is a rare cause of congenital adrenal insufficiency and is caused by the mutation of the genes responsible for steroidogenesis or adrenal cortex development. Three genes have been identified among the five major genetic causes of AHC [2]. Among the different genetic mutations, DAX1 deficiency due to genetic defects in NR0B1 is the commonest [1]. Regardless of the underlying genetic etiology, AHC is associated with a deficiency of all adrenocortical hormones. Rapid and life-threatening deterioration of adrenal function may occur, and early diagnosis with early initiation of mineralocorticoid and glucocorticoid treatment prevents acute adrenal crisis. In this presentation, we have reviewed five patients diagnosed with congenital adrenal insufficiency other than CAH.

## Case presentation

Patient information

Between 2010 and 2019, four boys and one girl were diagnosed by us with congenital primary AI, other than CAH. All patients were of local South Asian origin and were born of non-consanguinous parents. The clinical and laboratory data are summarized in Table 1. In all cases, both parents gave informed consent for the collection of genetic material from their children and for publication of their records. The family of two patients gave permission to reproduce patient photographs. There was no history of diabetes or gestational diabetes in any of the mothers.

**Table 1:** Presentation of different cases APC: adenomatous polyposis coli

Case number	1	2	3	4	5
Sex	Male	Male	Male	Male	Female
Birth history	Full-term cesarean delivery	Full-term cesarean delivery	Full-term cesarean delivery	Full-term cesarean delivery	Full-term normal vaginal delivery
Family history	No	No	No	No	Two older siblings died after trivial illnesses, and each one had progressive darkening of the skin
Age at the onset of symptoms	Fifth day	Sixth day	Fifth day	Seventh day	20th month
First symptom	Hypoglycemia	Hypoglycemia	Hypoglycemia	Hypoglycemia	Progressive darkening of the skin
Consanguinity	No	No	No	No	No
Genitalia	Normal male	Normal male	Normal male	Normal male	Normal female
FPG	28	28	28	32	88
Sodium (normal: 133-146 mEq/L)	131	120	123	126	126
Potassium (normal: 3.6-5.2 mEq/L)	6.8	6.2	7.4	6.7	5.4
17-OHP (normal: 3-90 ng/mL)	14.86	8.0	1.5	1.8	3.2
Cortisol (8 am) (normal: 5.50-19.8 μg/dL)	0.39	1.9	Undetectable	1	Undetectable
Cortisol (post-Synacthen) (normal: >18 μg/dL)	Not done	Not done	1	2	Not done
ACTH (normal: 10-88 pg/mL)	316	377	236	200	240
USG: adrenal	Not done	Normal	Normal	Normal	Not done
CT: adrenal	Normal	Not done	Not done	Not done	Normal
Clinical exome analysis	No NR0B1/DAX1 or NR5A1 (SF1) or AAAS mutations found	APC mutation found but no NR0B1/DAX1 or NR5A1 (SF1) or AAAS mutations found	No NR0B1/DAX1 or NR5A1 (SF1) or AAAS mutations found	No NR0B1/DAX1 or NR5A1 (SF1) or AAAS mutations found	AAAS mutations found
Management	Oral hydrocortisone and fludrocortisone	Oral hydrocortisone and fludrocortisone	Oral hydrocortisone and fludrocortisone	Oral hydrocortisone and fludrocortisone	Oral hydrocortisone and fludrocortisone

All patients had routine blood biochemistry, followed by measurement of baseline cortisol, 17-hydroxyprogesterone (17-OHP), and ACTH. The post-ACTH measurement of cortisol and 17-OHP was done in two patients. The ACTH test was performed after 24-hour discontinuation of treatment, with an intravenous bolus injection of 125 μg tetracosactide (Synacthen). Blood samples were collected in prechilled heparinized tubes before and 30 minutes after the injection, centrifuged at 4C, and stored frozen until assayed. The test could not be performed on the other three patients due to a worldwide shortage of tetracosactide. As four out of five patients presented with neonatal hypoglycemia, the standard endocrine workup for hypoglycemia was performed for them. This included a collection of blood in the critical period (of hypoglycemia) for cortisol, free thyroxine (FT4), thyroid-stimulating hormone (TSH), growth hormone (GH), insulin, and C-peptide. Once cortisol deficiency was identified, ACTH, cortisol, and 17-OHP were tested. As patient 5 presented with increased pigmentation, she was tested for cortisol, ACTH, and 17-OHP only apart from routine biochemistry.

Golden Helix VarSeq 2.2.0, the clinical genomics interpretation and reporting platform from Golden Helix Inc. (Bozeman, MT, United States), was used for genetic study result analysis. The variant annotation engine includes algorithms to identify the variant impact on genes using both public content ClinVar, HPO, links to dbSNP, gnomAD, and in silico predictors - GERP++, PhyloP, PhyloP LRT, SIFT, and PolyPhen2. VarSeq allows quick filtering and evaluation of variants. Clinically relevant variants were annotated using published variants in literature and a set of disease databases - dbSNP, ClinVar, OMIM, and HGMD. Common variants were filtered based on allele frequency in gnomAD. Only non-synonymous and splice site variants found in the clinical exome panel consisting of a specific set of genes were used for clinical interpretation. The genetic test results were reported based on the recommendations of the American College of Medical Genetics [3].

Four out of the five patients had a common clinical presentation in that they presented with hypoglycemia between the fifth and seventh days of life. All had uneventful deliveries, four by cesarean section (CS), and all were morphologically normal.

Case 1

**Figure 1:**
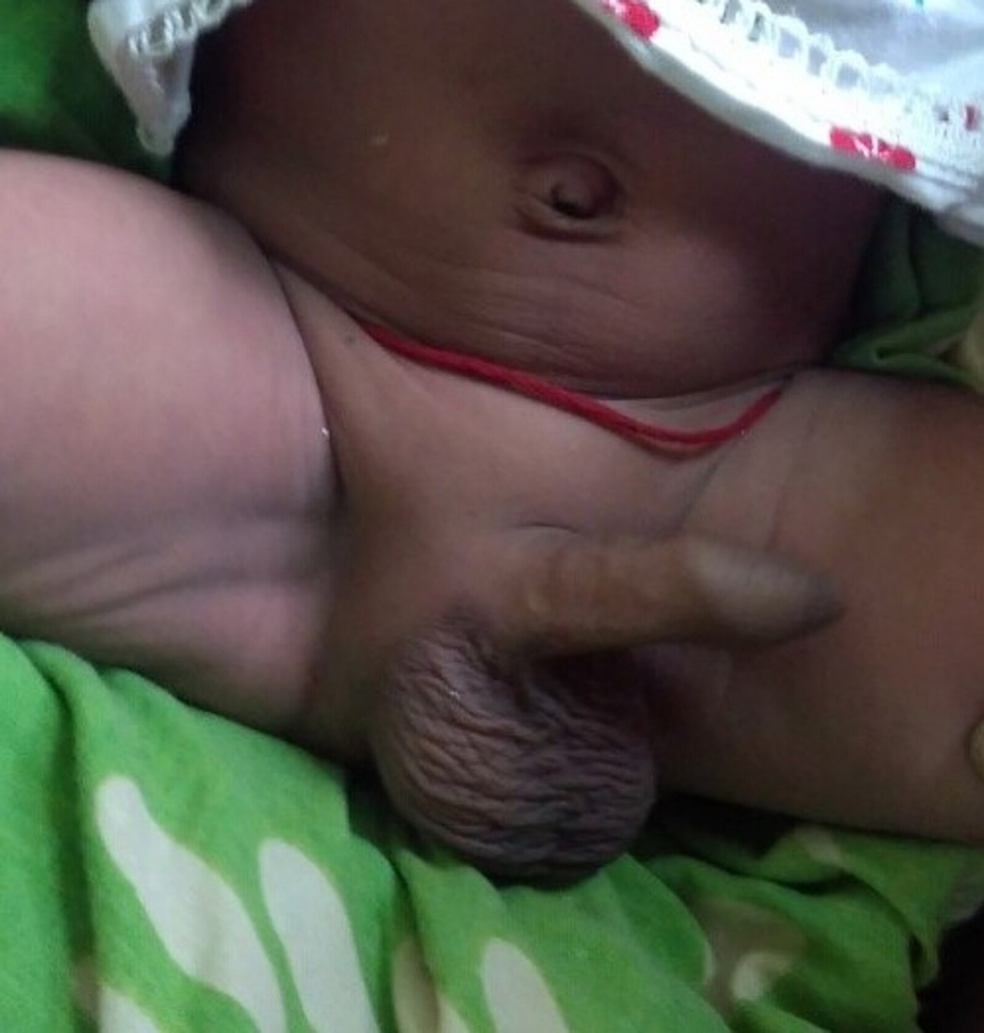
Case 1 Male baby without any abnormality in the external genitalia

The male baby had a term CS delivery; birth weight was 2.2 kg, with an Apgar score of 10/10 at five minutes. The baby was breastfed and did well until the fifth day when he had hypoglycemia (capillary blood glucose was 28 mg/dL). Hypoglycemia was confirmed by the laboratory and was treated with intravenous (IV) glucose. Subsequently, hypoglycemia was allowed to develop, and blood was drawn for the standard tests (glucose, insulin, C-peptide, GH, FT4, TSH, and cortisol) during the critical period. Plasma cortisol was very low (0.4 µg/dL), ACTH was elevated (316 pg/mL), and 17-OHP was normal (14.86 ng/mL). A plain computed tomography (CT) scan of the abdomen revealed morphologically normal adrenal glands. The baby was started on oral hydrocortisone and fludrocortisone and has been well since. Later, genetic studies excluded the common mutations associated with adrenal deficiency (Figure 1).

Case 2

The male baby was born by CS at term with normal Apgar. The presentation was similar to case 1 in that hypoglycemia occurred on day 6. Later, blood sampling during the critical period established the diagnosis of AI. Genetic studies were negative for the common mutations associated with AHC but revealed an unrelated familial adenomatous polyposis coli (APC) gene mutation. The baby was started on oral hydrocortisone and fludrocortisone. Genetic counseling and testing for APC gene mutation were offered to the family members of the baby.

Case 3

The male baby was born by CS at term with normal Apgar. Hypoglycemia occurred on the fifth day. Diagnosis and management were similar to the earlier two patients. Genetic studies were similarly negative for the target genes. Later in the fifth month, hydrocortisone was withheld for one day, and a short Synacthen© test was done, which showed persistence of hypocortisolemia. At present, he is 36 months old and has growth retardation. A magnetic resonance imaging (MRI) of the head showed mild Arnold Chiari malformation for which no treatment was recommended by the neurology service. His parents declined a workup for growth retardation.

Case 4

The male baby was born by CS at term with normal Apgar. He presented on the seventh day of life with hypoglycemia. Work up and management were similar to the previous three cases. Later in the sixth month, hydrocortisone was withheld for one day, and a short Synacthen© test was done, which showed persistence of hypocortisolemia. Genetic studies were negative for the target mutations.

Case 5

**Figure 2:**
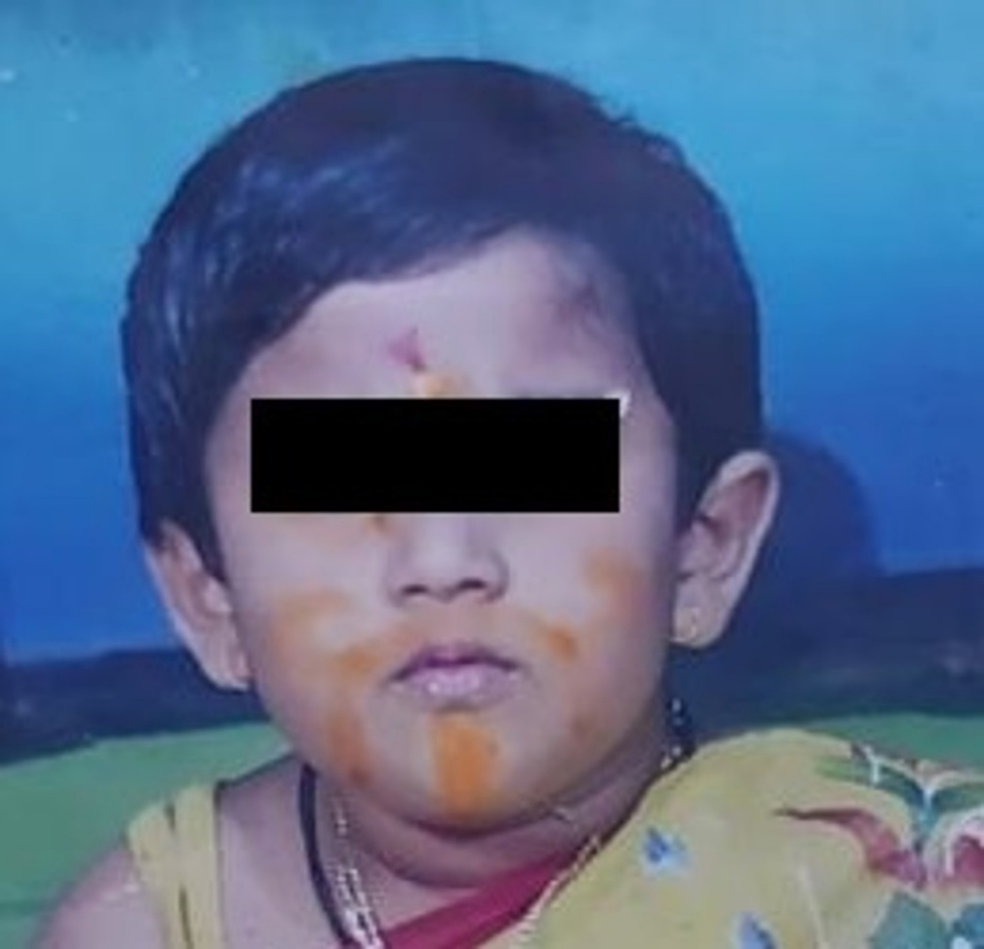
Case 5 (on presentation) Female child on presentation with darkening of the skin

**Figure 3:**
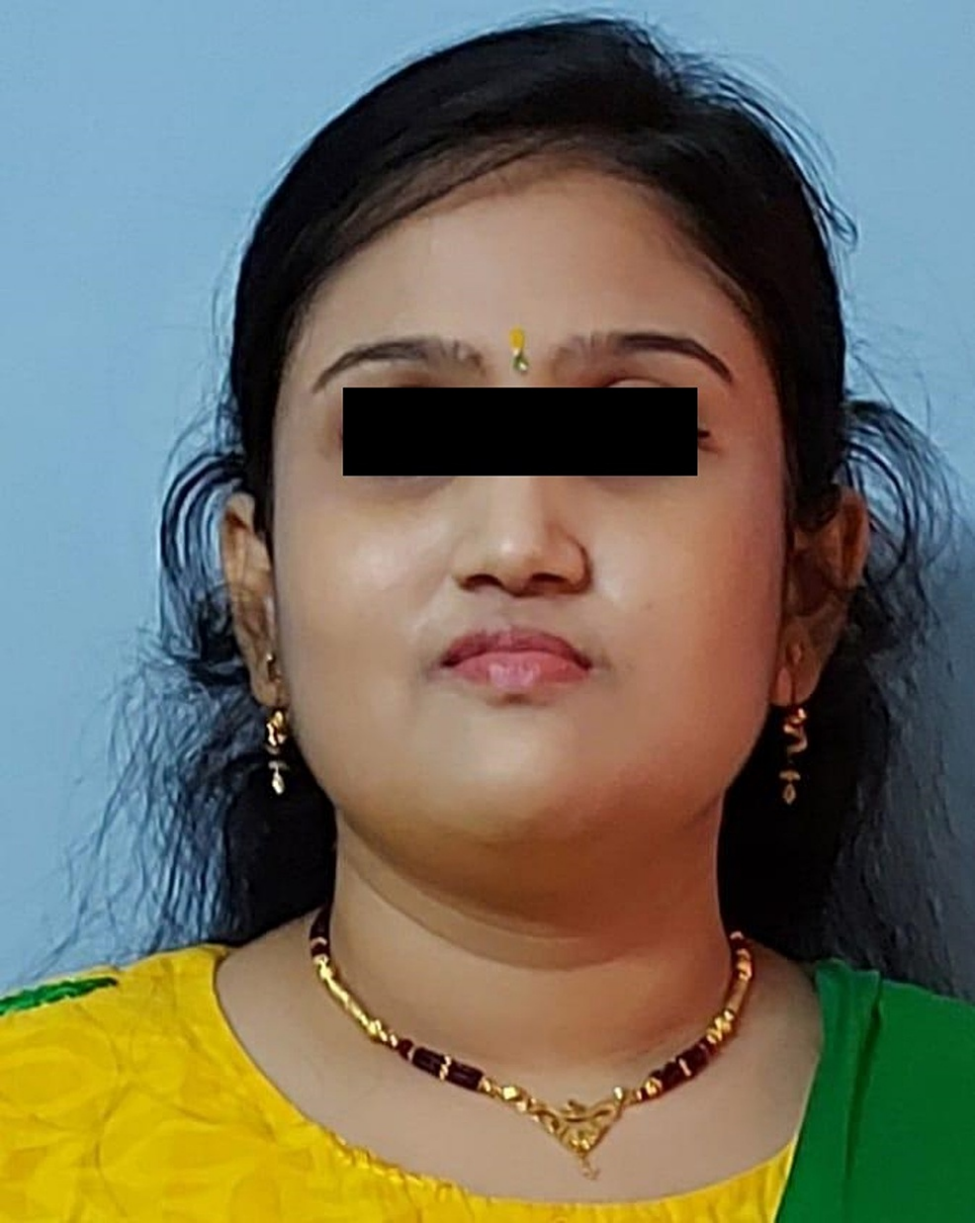
Case 5 (after steroid replacement) Female child with significant improvement in the darkening of the skin after glucocorticoid replacement for eight years

The female child presented at 20 months of age. She was born at term by normal vaginal delivery. The neonatal period and early infancy were unremarkable. The reason for consultation was a progressive darkening of her skin (Figure 2). The parents reported that she had two older siblings who developed similar darkening of the skin, and both died after trivial illnesses. She was found to have undetectable cortisol, a high ACTH level, and normal 17-OHP. Genetic studies showed mutation associated with the AAAS gene. Other classic phenotypes of triple A syndrome (alacrima and achalasia) were not present at the time of presentation but developed post-puberty. She did not exhibit any signs of autonomic dysfunction (pupillary abnormalities, abnormal sweating, orthostatic hypotension, or disturbances of heart rate). She is doing well on hydrocortisone and fludrocortisone replacement (Figure 3).

Results

We diagnosed five cases of congenital adrenal hypoplasia. All are doing well with glucocorticoid and mineralocorticoid replacement. Commercially available genetic studies were done on all the patients. Only one patient (patient 5) had a recognized mutation in the AAAS gene. One patient (patient 2) had a nonspecific APC mutation. In patients 1 to 4, we did not find any of the recognized (NR0B1/DAX1 mutation, NR5A1/SF1 mutation, or AAAS gene) mutation for AHC.

## Discussion

AHC was first described by Šikl in 1948 [4]. The clinical features, hormonal findings, and diagnostic criteria are well defined, and the gene mutations responsible have been identified, albeit in a Western population. The condition has been rarely reported in South Asia. Consequently, there has not been an opportunity to perform genetic analysis in a South Asian population. Our experience with four recent patients shows that the mutations responsible for AHC in this population are different from what has been described in Western populations.

The diagnosis of primary adrenal insufficiency was confirmed by low plasma cortisol (less than 5 μg/dL at 8 am) in combination with plasma ACTH value more than twofold the upper limit of the reference range. Autoimmune AI does not develop from birth, and no autoimmune evaluation was done. The possibility of two other causes of congenital adrenal insufficiency (CAH and con­genital lipoid adrenal hyperplasia) [2] was ruled out, as the adrenal glands were not enlarged, and the 17-OHP levels were normal in all five cases. The signs and symptoms in infants with AHC include recurrent hypoglycemia, hyponatremia, hyperkalemia, and hyperpigmentation with normal external genitalia [5]. The findings are characteristic of combined glucocorticoid and mineralocorticoid deficiencies. Most patients present with a salt-losing crisis in the first week of life [5]. In our experience, this was not the case. Four of our patients presented with neonatal hypoglycemia, and one had progressive skin pigmentation. It appears that cortisol deficiency leading to hypoglycemia preceded aldosterone deficiency in our series. In contrast, neonates with a commonly described DAX-1/NROB1 gene mutation in the Western subset present with salt-wasting rather than with hypoglycemic symptoms indicating predominant aldosterone deficiency [5].

There are five major genetic causes of AHC: 1) X-linked AHC (gene: DAX-1/NROB1, chromosomal location: Xp21.3-p21.2), 2) SF-1-linked AHC (gene: SF-1/NR5A1, chromosomal location: 9q33), 3) autosomal recessive AHC (gene: unknown), 4) IMAGe syndrome (gene: unknown), 5) ACTH insensitivity: triple A syndrome (gene: AAAS, chromosomal location: 12q13) (Table 2). The three commonly described genes (NR0B1/DAX1, NR5A1/SF1, and AAAS mutations) of AHC were evaluated in all five cases. AAAS gene mutation was found in case 5. Mutations of the three commonly associated genes were not found in the other four cases. DAX-1(NR0B1) gene mutation is responsible for X-linked AHC. As DAX-1 plays an important role in the development and function of the adrenal, as well as the hypothalamic-pituitary-gonadal axis, males affected with DAX-1(NR0B1) gene mutation also suffer from hypogonadotropic hypogonadism and cryptorchidism due to selective deficiency of gonadotropins (LH and FSH) [5]. However, the production of other pituitary hormones (ACTH, GH, TSH, and PRL) remain normal [5]. All boys with AHC with DAX-1(NR0B1) gene mutation develop delayed puberty due to hypogonadotropic hypogonadism [5] and needs close follow-up during pubertal age. None of our four male infants had cryptorchidism nor DAX-1(NR0B1) gene mutation. SF-1 is involved in the regulation of steroidogenesis, and SF-1 gene mutation causes adrenal insufﬁciency and XY sex reversal (46, XY karyotype, normal female external genitalia, streak gonads, azoospermia, and the presence of normal Müllerian structures) in a phenotypically female child [2]. The lone female infant did not show SF-1 mutation but had an AAAS mutation. Mutations in the AAAS gene cause triple A syndrome (Allgrove syndrome), which is an autosomal recessive condition characterized by ACTH-resistant adrenal insufficiency and classically associated with reduced or absent tearing (alacrima) and achalasia [6]. Triple A syndrome is an extremely heterogeneous multisystem disorder with endocrine, gastrointestinal, ocular, and neurological manifestations [6]. IMAGe syndrome is a rare congenital disorder and is characterized by intrauterine growth restriction (IUGR), metaphyseal dysplasia, adrenal hypoplasia congenita, and genitourinary abnormalities (cryptorchidism, micropenis, hypospadias, and chordee in males) [7]. Diagnosis of this syndrome was not considered, as none of the four male infants had any feature suggestive of IMAGe syndrome. Hence, case 5, a female infant, was suffering from triple A syndrome, and all other four male infants (case 1 to 4) were suffering from autosomal recessive AHC due to yet unknown mutations. Unusually, the X-linked AHC with a genetic mutation at DAX-1/NROB1, the commonest variety, was absent in our series of five cases. An unrelated APC gene mutation was incidentally observed in case 2. Adenomatous polyposis coli (APC) gene is associated with familial adenomatous polyposis (FAP), an autosomal dominant syndrome [8]. The syndrome is associated with hundreds to thousands of polyps developing throughout the colon and rectum from the early teenage years with a nearly 100% lifetime risk of colorectal cancer. The presence of this incidental finding and the possible consequence were discussed with the parents of the boy.

**Table 2:** Molecular genetics of AHC

Disorder	Gene	Chromosomal location
X-linked AHC	DAX-1/NROB1	Xp21.3-p21.2
SF-1 linked AHC	SF-1/NR5A1	9q33
Autosomal recessive AHC	Unknown	-
IMAGe syndrome	Unknown	-
ACTH insensitivity: triple A syndrome	AAAS	12q13

## Conclusions

AHC mostly presents with hypoglycemia without any genital abnormality or any structural abnormality of the adrenal gland. Glucocorticoid and mineralocorticoid replacement is lifesaving. The pathophysiology of AHC is not well understood. It is a genetic disorder with recognized mutations in most cases. However, in four out of our five cases, none of the recognized mutations were present, although clinically and biochemically all our patients had AHC. This leads to the conclusion that there are hitherto unidentified mutations responsible for AHC in the South Asian population.
